# Possible role of *SCN4A* skeletal muscle mutation in apnea during seizure

**DOI:** 10.1002/epi4.12347

**Published:** 2019-07-01

**Authors:** Dilşad Türkdoğan, Emma Matthews, Sunay Usluer, Aslı Gündoğdu, Kayıhan Uluç, Roope Mannikko, Michael G. Hanna, Sanjay M. Sisodiya, Hande S. Çağlayan

**Affiliations:** ^1^ Medical Faculty, Department of Child Neurology Marmara University Istanbul Turkey; ^2^ Queen Square Centre for Neuromuscular Diseases, UCL Queen Square Institute of Neurology UCL and National Hospital for Neurology and Neurosurgery London UK; ^3^ Formerly Affiliated with Department of Molecular Biology and Genetics Boğaziçi University Istanbul Turkey; ^4^ Department of Molecular Biology and Genetics Boğaziçi University Istanbul Turkey; ^5^ Medical Faculty, Department of Clinical Neurophysiology and Neurology Marmara University Istanbul Turkey; ^6^ Department of Clinical and Experimental Epilepsy UCL Queen Square Institute of Neurology London UK; ^7^ Chalfont Centre for Epilepsy Bucks UK; ^8^ İzmir Biomedicine and Genome Center İzmir Turkey

**Keywords:** laryngospasm, SUDEP, myotonia, sodium channel

## Abstract

*SCN4A* gene mutations cause a number of neuromuscular phenotypes including myotonia. A subset of infants with myotonia‐causing mutations experience severe life‐threatening episodic laryngospasm with apnea. We have recently identified similar *SCN4A* mutations in association with sudden infant death syndrome. Laryngospasm has also been proposed as a contributory mechanism to some cases of sudden unexpected death in epilepsy (SUDEP). We report an infant with EEG‐confirmed seizures and recurrent apneas. Whole‐exome sequencing identified a known pathogenic mutation in the *SCN4A* gene that has been reported in several unrelated families with myotonic disorder. We propose that the *SCN4A* mutation contributed to the apneas in our case, irrespective of the underlying cause of the epilepsy. We suggest this supports the notion that laryngospasm may contribute to some cases of SUDEP, and implicates a possible shared mechanism between a proportion of sudden infant deaths and sudden unexpected deaths in epilepsy.

## 1 INTRODUCTION

The *SCN4A* gene codes for the alpha sub‐unit of the voltage‐gated sodium channel Nav1.4, which is essential for muscle membrane excitability and contraction.^1^ It is the only sodium channel isoform expressed in adult skeletal muscle. Mutations of *SCN4A* are consequently associated with a range of neuromuscular phenotypes without systemic involvement, including autosomal‐dominant myotonia, and/or periodic paralysis, and autosomal recessive congenital myasthenia and congenital myopathy.^1^, ^2^, ^3^ Respiratory and laryngeal muscle compromise is common in affected infants and children and can cause life‐threatening respiratory impairment including recurrent apneas.^4^ The autosomal‐dominant *SCN4A* disorders such as myotonia are episodic. Infants with *SCN4A*‐related myotonia can appear outwardly healthy but present acutely with recurrent episodes of generalized stiffening of the trunk and limbs, apnea, and cyanosis (due to respiratory and laryngeal muscle myotonia‐causing laryngospasm) which may be accompanied by bradycardia and loss of consciousness. This specific myotonic phenotype has been named severe neonatal episodic laryngospasm^5^ (SNEL). An erroneous diagnosis of generalized epilepsy is frequently made in these infants.^6^ Events can be life‐threatening or “near miss” with several infants requiring ventilation and prolonged ITU admissions.^5^, ^7^ Such acute life‐threatening events recently led us to investigate *SCN4A* gene mutations in cases of sudden infant death. We found a small but significant proportion of cases carried rare functionally deleterious variants. Laryngospasm has also been proposed as a contributory mechanism in sudden unexpected death in epilepsy (SUDEP).^9^, ^10^, ^11^, ^12^

Here, we report a child who was investigated and treated for generalized seizures associated with apneic spells and was found to carry the heterozygous p.A1156T *SCN4A* mutation. This mutation has been reported in several independent families with myotonia, and functional studies have confirmed its pathogenicity.^13^, ^14^, ^15^ We hypothesize that *SCN4A* mutation may contribute to apnea during the physiological stress of seizures.

## 2 CASE REPORT

**Figure 1:**
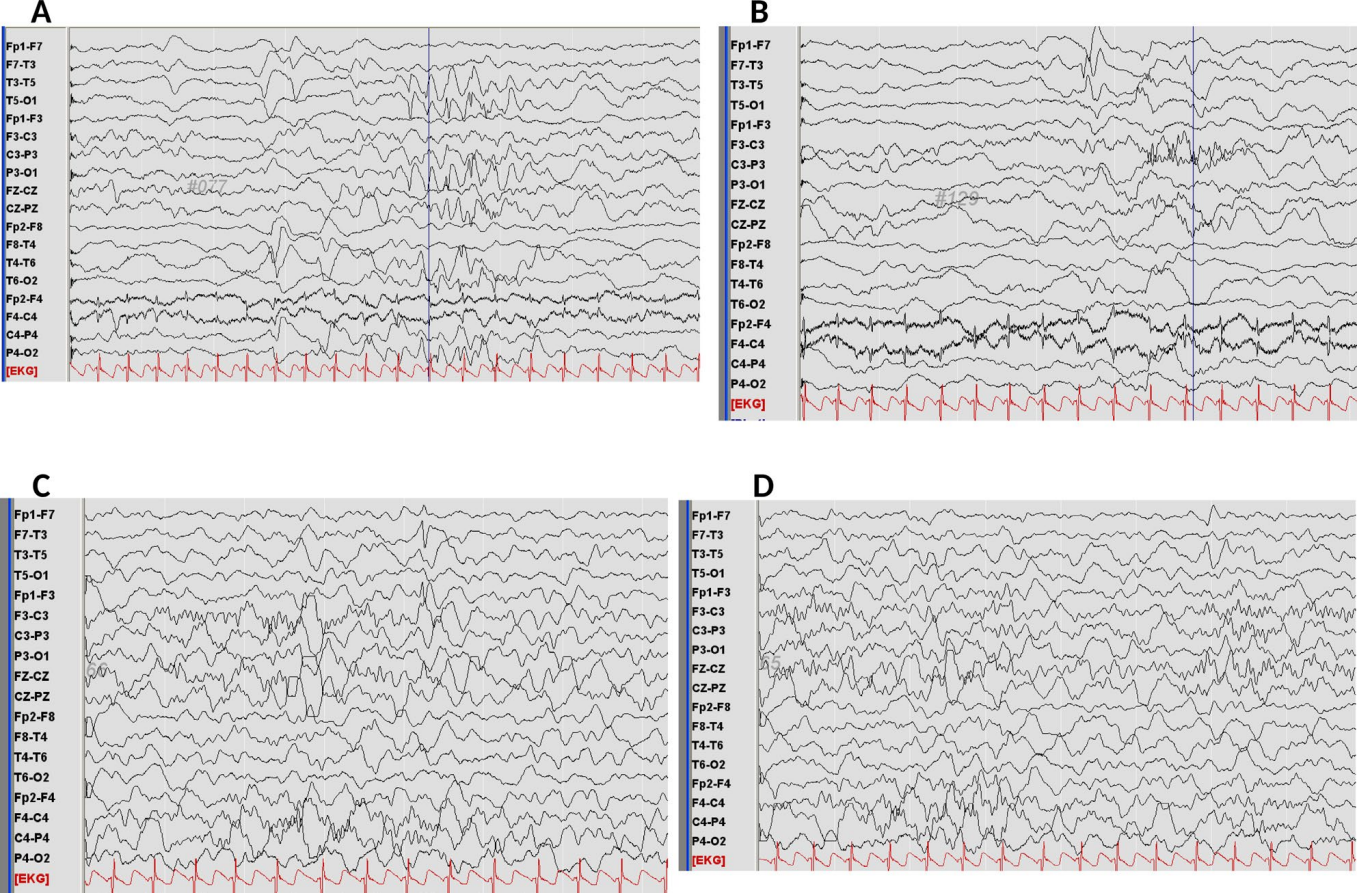
EEG examination at 2 months of age showed repetitive sharp waves at left occipital region (A) and left temporal region (B). EEG examination at 3 months of age showed sharp waves at left frontal (C) and left temporal region (D)

Written informed consent was obtained from the family for this case study. A male infant presented with episodes beginning at 35 days of age that were triggered by feeding and characterized by facial flushing, apnea, and cyanosis followed by stiffening of the lower limbs lasting up to 20 seconds. A cluster of six similar events, all with apnea, and reported by his mother, occurred over three consecutive days. Pre‐, peri‐, and post‐natal history was unremarkable. He had normal neurological examination at 39 days of age. Cardiac evaluation including echocardiogram and ECG was also normal. He was admitted for further investigation and telemetry. EEG examination captured a focal seizure characterized by opening of the eyes, version of the head to the left side, flushing and evolving cyanosis of the face with tonic contraction of the lower extremities. Ictal EEG activity lasting 15 seconds originated from the left temporal region. Serial interictal EEG examinations done at two and three months of age showed multifocal sharp waves from the left hemisphere (Figure 1). Following phenobarbital loading, the seizures immediately resolved. Metabolic screening and cranial MRI were unremarkable. Medical therapy was continued for 13 months and then stopped, and no seizures have recurred.

In the subsequent follow‐up period, the child, currently nine years old, has been generally well with normal developmental milestones. No muscle weakness, cramps, or myotonia‐like symptoms have been reported. Neurological, including neuromuscular, examination and academic skills have been within normal limits except for mild attention deficit disorder. The only family history reported was of the elder brother having a single febrile seizure. As no specific cause for the seizures was identified, genetic investigations were undertaken.

### 2.1 Methods

DNA was extracted from 10 mL peripheral blood using standard procedures. Coding sequences and exon/intron boundaries of two candidate genes, *SCN2A* and *KCNQ2,* were amplified by PCR as described previously^16^ and Sanger‐sequenced. Whole‐exome sequencing was performed using the NimbleGen EZ Whole Exome Enrichment Kit on the Illumina HiSeq2000 platform with 100X coverage. Sequence annotation and variant calling were done on Genomize SEQ platform (https://seq.genomize.com), and variant prioritization was done at MAF <1% in all normal populations. ClinVar associations and all destructive and missense variants were filtered from genes in Epilepsy_HPO_September 2018 and/or MORBID OMIM May 2018. PCR and Sanger sequencing were performed to confirm the *SCN4A* c.G3466A nucleotide change in the proband and for segregation analysis in the family using primers: F: 5′‐CCCACGTTGTCGTAGTTGAC‐3′ and R: 5′‐TGGGTGGCGTAGAGATGTGG‐3′.

### 2.2 Results

**Figure 2:**
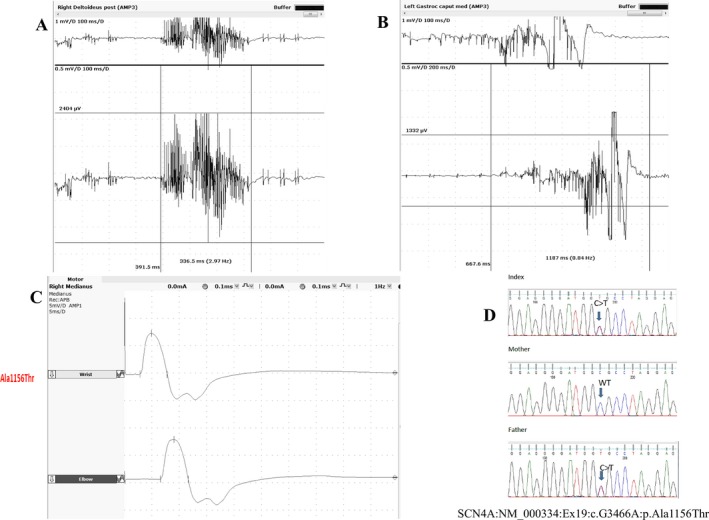
EMG examinations done in the father carrying the *SCN4A* variant showed increased insertional activity on upper (A) and lower extremity muscles (B) and delayed lower amplitude motor response following the compound motor action potential on median motor NCS (C) and Sanger sequencing results of the *SCN4A* c.G3466A variant in the family (D)

Candidate gene and WES analysis of epilepsy genes and others expressed in the brain did not demonstrate any known pathogenic or potentially pathogenic variants. Filtering for ClinVar associated variants in MORBID OMIM 2018 revealed a previously reported heterozygous *SCN4A* variant c.G3466A, p.A1156T.^12^, ^13^ This variant was inherited paternally and confirmed in the proband's father (Figure 2D). The father denied any symptoms of myopathy and/or myotonia, and none were demonstrated on clinical examination. The father's nerve conduction study parameters of latency, amplitude, and velocity were found to be within normal limits, but delayed lower amplitude motor responses following the compound motor action potential, indicating post‐exercise myotonic potentials, were observed (Figure 2C). Needle EMG revealed myotonic potentials lasting more than 300 milliseconds in all sampled muscles (Figure 2A,B). The child's parents did not wish him to undergo an EMG examination.

## 3 DISCUSSION

Several cases of infantile *SCN4A* myotonia with laryngospasm and apnea have been reported to be erroneously diagnosed as generalized seizures, delaying appropriate therapy.^6^ We initially re‐examined the clinical, EEG, and WES data from this case with this possibility in mind. However, EEG clearly demonstrated epileptiform discharges during symptoms and interictally. The WES data confirm the infant does carry a paternally inherited known pathogenic *SCN4A* mutation, shown previously to be functionally deleterious, with neurophysiological evidence of myotonia in the father. The p.A1156T mutation has been described to cause myotonia and periodic paralysis with variable severity in several unrelated families. The largest series described several cases in which myotonia or increased insertional activity was only detected by neurophysiology, without overt clinical symptoms, and in others who complained of more obscure symptoms of myalgia.^13^ This is compatible with the history in the father of our proband; he reported no significant clinical symptoms but had EMG evidence of myotonia, an abnormal finding that supports a myotonic disorder. Functional studies have confirmed a clear deleterious impact of this mutation on channel function, with impaired fast inactivation and accelerated recovery from inactivation.^13^ A gain‐of‐function effect mediated via impaired fast inactivation is the hallmark of all *SCN4A* myotonia‐causing mutations.^1^ The p.A1156T mutation is present at low frequency (15 individuals) in the gnomAD database. However, considering that this variant can be associated with neurophysiology evidence of myotonia without overt clinical symptoms (as is the case of the proband's father), asymptomatic cases might have been included in large population datasets, such as gnomAD. It has been clearly established to cause myotonia but the variable clinical expression suggests other genetic and epigenetic factors may contribute to overall severity.

We recently described a similar gain‐of‐function mutation in sudden infant death,^8^ and such mutations are a recognized cause of recurrent life‐threatening apneas in infants with myotonia and laryngospasm.^4^, ^5^ In children with the latter, there is clear evidence that the muscle phenotype evolves with age; that is, apneas are prominent and symptomatic in early life but diminish with age.^6^

The clinical presentation of infantile myotonia with laryngospasm can easily be mistaken for generalized epilepsy.^6^ Myotonia is a crucial diagnosis to make as it is eminently treatable with sodium channel blockers. We would advocate that myotonia be at least considered in the differential diagnosis of infants presenting with recurrent events with associated apnea in the presence or absence of telemetry‐proven seizures. We propose that our case illustrates a further clinical scenario, of epileptic seizures of unknown cause, with a genuine comorbidity (apnea likely related to mutation in *SCN4A*).

Apnea and cyanosis can be an independent consequence of seizures, and we cannot definitively state that the *SCN4A* mutation in this case caused laryngospasm and contributed to the episodic apnea, and we do not suggest that the *SCN4A* mutation was the cause of the proband's epilepsy per se. We propose, however, that SCN4A mutations may contribute to apnea in infants during seizures of whatever cause. Recently, laryngospasm and upper airway obstruction have been shown to contribute to hypoxia and death in rat models of sudden unexpected death in epilepsy.^9^, ^12^ There are also cases of ictal and post–ictal laryngospasm contributing to apnea and “near miss” events requiring intubation in adults with refractory epilepsy,^10^, ^11^ further supporting the notion that laryngospasm may contribute to some cases of sudden unexpected death in epilepsy (SUDEP). This suggests there may be a shared mechanism of upper airway obstruction between some cases of sudden infant death and sudden unexpected death in epilepsy, a hypothesis requiring further testing.

There has been one other relevant case published of which we are aware. A rare *SCN4A* variant of uncertain significance has been reported in an individual with generalized epilepsy and a family history (deceased mother) of probable SUDEP.^17^ The variant was not present in the proband's father but the mother's DNA was not tested.

Although often considered to be tissue‐specific, the expression pattern of sodium channel isoforms varies throughout life. In adult skeletal muscle, Nav1.4 is the only isoform present but in infancy the cardiac muscle isoform Nav1.5 is also expressed in skeletal muscle. Nav1.5 expression is downregulated during development but upregulated in denervated adult muscle. Conversely cardiac muscle expresses Nav1.4 and Nav1.5 for a limited period, before Nav1.5 becomes the sole channel expressed. There is also evidence of the presence of Nav1.4 in the mouse and human brain, although relative expression levels at different stages of life are unknown. In a family with a function‐changing Nav1.4 mutation and CNS symptoms of tremor and epilepsy, the authors postulated this expression could increase the susceptibility to epilepsy.^18^ However, *SCN4A* mutations have so far not been associated with epilepsy pathogenesis.

In summary, we report a pathogenic *SCN4A* mutation, previously associated with myotonia, in a child with epilepsy and concurrent apneas. We propose the *SCN4A* mutation may contribute to the apneas via laryngeal and respiratory muscle myotonia. This is consistent with the notion that laryngospasm may contribute to some cases of sudden unexpected death in epilepsy (SUDEP) and may suggest a shared mechanism between some cases of sudden infant death and SUDEP. Our findings need exploration in other cohorts of patients who have succumbed to SUDEP, or have documented apnea or laryngospasm, irrespective of the putative cause of the epilepsy in such patients. *SCN4A* mutations may constitute genetic comorbidity, increasing risks of laryngospasm and attendant mechanical apnea during epileptic seizures. More data are required to determine this and any implications for the treatment and monitoring requirements of such patients.

## CONFLICTS OF INTEREST

EM has received an honorarium for attending an advisory board organized by LUPIN pharmaceuticals. The remaining authors have no conflicts of interest. We confirm that we have read the journal's position on issues involved in ethical publication and confirm that this report is consistent with those guidelines.
